# History of malaria research and its contribution to the malaria control success in Suriname: a review

**DOI:** 10.1186/1475-2875-11-95

**Published:** 2012-03-29

**Authors:** Florence JV Breeveld, Stephen GS Vreden, Martin P Grobusch

**Affiliations:** 1 Center for Tropical Medicine and Travel Medicine, Department of Infectious Diseases, Division of Internal Medicine, Academic Medical Center, University of Amsterdam, Meibergdreef 9, PO Box 22660, 1100 DE Amsterdam, The Netherlands; 2 Academisch Ziekenhuis Paramaribo, Paramaribo, Suriname; 3 Institute of Tropical Medicine, University of Tübingen, Tübingen, Germany

**Keywords:** Artemisinin-based combination therapy, Control, Elimination, Eradication, Gold mining, Malaria, Resistance, Suriname

## Abstract

Suriname has cleared malaria from its capital city and coastal areas mainly through the successful use of chloroquine and DDT (dichloro-diphenyl-trichloroethane) during the Global Malaria Eradication programme that started in 1955. Nonetheless, malaria transmission rates remained high in the interior of the country for a long time. An impressive decline in malaria cases was achieved in the past few years, from 14,403 registered cases in 2003 to 1,371 in 2009. The introduction of artemisinin-based combination therapy (ACT) in 2004 has further fuelled the decrease in the number of infections with *Plasmodium falciparum*. The only population group still heavily burdened with malaria is gold mining industry workers. Interestingly, an important part of malaria cases diagnosed and treated in Suriname originate from border regions. Therefore, practical initiatives of combined efforts between neighbouring countries must be scaled up in order to effectively attack these specific areas. Furthermore, it is of vital importance to keep investing into the malaria control programme and public awareness campaigns. Especially the correct use of ACT must be promoted in order to prevent the emergence of resistance. However, effective preventive measures and adequate therapeutic options are on their own not enough to control, let alone eliminate malaria. Changing personal and social behaviour of people is particularly difficult, but crucial in making the current success sustainable. With this in mind, research on successfully implemented interventions, focusing on behavioural modifications and methods of measuring their effectiveness, must be expanded.

## Background

**Figure 1:**
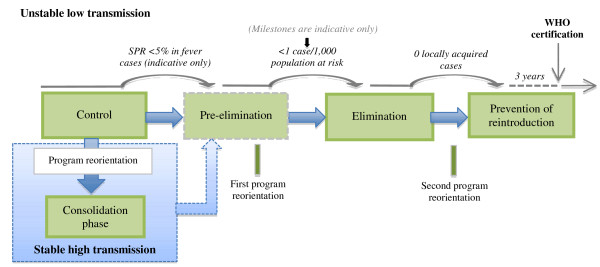
**The malaria - control to elimination - continuum **[4].

**Figure 2:**
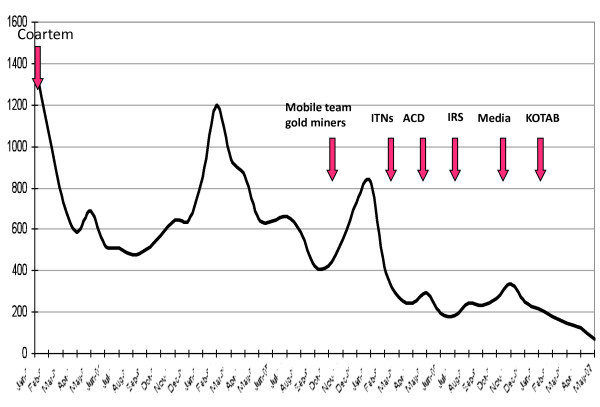
**Implementation of malaria interventions in Suriname, 2004 - 2007**. See list of acronyms for abbreviations.

The number of estimated direct deaths due to malaria worldwide has decreased from 985,000 in 2000 to 781,000 in 2009 [1]. Although malaria remains a major health burden in tropical and subtropical countries; with the majority of cases in sub-Saharan Africa, several regions show an impressive decline of malaria cases and a lower number of malaria-associated deaths. A renewed global interest in malaria eradication and increased international funding has boosted malaria control efforts all over the world. The elimination of malaria from low-transmission areas seems feasible. Throughout history, Suriname is one of the countries that have effectively decreased the incidence of malaria cases within its borders. Impressively, it has reduced the number of registered malaria cases from 14,403 in 2003 to 1371 in 2009, from which 689 were confirmed by microscopy and 682 by rapid diagnostic testing (RDT) [2]. This achievement was internationally acknowledged and rewarded in November 2010, when the National Malaria Board in Suriname was awarded the title "*Malaria Champion of the Americas*". Certain anti-malarial measures are known to be effective in malaria transmission areas throughout the world, but in order to move towards a stable situation of control and after that to the elimination [3,4] of malaria, country- and region-specific tools need to be implemented (Figure 1). Which anti-malarial interventions have made Suriname the success story in malaria control that it is acknowledged to be (Figure 2)? How can this help other countries in achieving similar results in decreasing malaria incidence? Knowledge of historical developments in malaria research and epidemiology in Suriname can provide guidance for the future, and possibly for other countries, too. Therefore, a review of the literature, addressing past and current difficulties and possibilities in malaria research, is relevant in assisting to pave the way towards further improved malaria control, if not regional elimination of the disease.

## Methods

A literature search was conducted between February and April 2011 to identify studies concerning historical developments in malaria research and how they affected malaria epidemiology in Suriname. Through a PubMed Query and searching the Cochrane systematic review database, using free text words and MeSH search strategy with the terms: 'malaria', 'epidemiology' and 'Suriname', studies published in English and Dutch were included. Furthermore, reference lists of original articles were reviewed to search for more relevant literature. In addition, information from local contacts in the capital and interior was gathered, illustrating firsthand experiences with the developments in malaria research specifically concerning the situation in Suriname.

## Results

### Historical background

The first malaria in Suriname was most likely imported through enslaved people that were shipped from western Africa around the year 1620. In the following years, many missionaries died from symptoms typical for '*malaria tropica'*. Reports going back to the late 18 hundreds describe the potential danger that was associated with visiting the interior of Suriname [5]. As will be discussed later, especially the border region between Suriname and French Guiana along the Marowijne (or Maroni) river is an area of concern with regard to malaria transmission. The region has been known to have the highest parasite index of the whole of South America [6,7]. The high burden of malaria in this region has been described decades ago. In 1888, Dr. J. Spitzly, chief of the military health service in Suriname, reported a case of *'fièvre paludienne fulminante'*, a very dangerous type of malaria, when visiting the hospital of St. Laurent du Maroni, the first settlement to be encountered when crossing the border river to French Guiana. He could not explain the major difference in malaria incidence that seemed to exist between the two border regions, other than that the river had its function in preventing transmission [8]. An explanation accepted later on was the existence of the so-called North-East Wind that presumably kept the *Anopheles *mosquitos away from Albina, a village in the far north-eastern part of Suriname. The French border was allegedly deprived from this wholesome wind because of its heavier forestation [5].

### El Niño southern oscillation

Even now in the 21^st ^century, numerous scientific opinions exist regarding the influences of weather and climate phenomena on the fluctuation of malaria incidence. Especially the El Niño Southern Oscillation (ENSO) is a widely discussed phenomenon. It consists of sea temperature changes in the Pacific Ocean (El Niño) and changes in atmospheric pressure across the Pacific Basin (the Southern Oscillation). These changes periodically have marked consequences for global weather and thus for public health, as they are associated with extreme weather like droughts, floods and storms [9]. Hanf *et al. *recently reported a positive diminishing influence of the ENSO on malaria incidence in French Guiana [10]. Although a statistical correlation was found, the actual predictive value of ENSO to modulate preventive measures aimed at reducing malaria incidence seems marginal in French Guiana. As for Colombia, the relation between ENSO and malaria is very strong [11]. The positive correlation can be used to predict high and low-risk years for malaria. This could provide the country with enough time to scale-up its anti-malarial strategies and, therefore, be prepared for an increase in malaria incidence. In Suriname however, no clear association has yet been found between the ENSO and yearly malaria incidence [9].

### Fighting the malaria burden

Paul C. Flu, Professor of Tropical Hygiene and Parasitology and former director of the Institute of Tropical Medicine in Leiden, the Netherlands, was the first Surinamese physician to diagnose *Plasmodium *species in Suriname in 1908. In one of his reports of a research trip to the country, he stated that one of the main reasons why Suriname could not bloom at that time, despite the rich natural resources and possibilities of the country, was the continuous burden of malaria, disabling people in their most productive years [12].

WHO launched the Global Malaria Eradication Programme (MEP) in 1955. In the following years, different programmes started to combat the enormous malaria burden existing at that time in Suriname. PAHO had started in 1952 with a programme to fight malaria and the Bureau for Public Health started the Anti-Malaria Campagne in 1957. The Malaria Eradication Programme attacked the disease on three major fronts [13]. Firstly, vector control was achieved through DDT (dichloro-diphenyl-trichloroethane) and/or dieldrin indoor residual spraying (IRS). All dwellings needed to be sprayed at regular intervals in order to kill all potentially infective malaria vectors. *Anopheles darlingi *was recognized as the most important malaria vector in the country [6,13]. This mosquito does not breed in the brackish water in the coastal areas where rivers from the inland meet the Atlantic Ocean. With IRS and control of potential breeding sites *An. darlingi *was eliminated from the coastal region. Secondly, regular collection of blood smears from the population for laboratory examination made the identification of malaria cases possible. The third major front in the attack was providing chemotherapy for every individual whose blood smear was found to be positive for malaria parasites. Providing medicated salt containing chloroquine to communities at risk was one of the strategies as well. These efforts resulted in the fact that neither Paramaribo nor the rest of the coastal area have reported any case of malaria transmission since 1960.

Although the capital city and the rest of the coastal area had been free of malaria transmission, the disease remained a major problem in the interior of the country. During the internal armed conflict of 1986-1989, a civil war between the National Army and guerrilla troops, access to the eastern part of Suriname was made impossible. Essential infrastructure - roads, bridges, airstrips - connecting the capital city Paramaribo with the eastern part of Suriname and neighbouring country French Guiana was blocked or demolished. Safety along the East-west connection and large areas in the interior depending on this main route could not be guaranteed and, therefore, movement of people and supplies was severely limited. These conditions disrupted the functioning of local health care facilities as well, which may explain the increase in the number of malaria cases at that time. The region has not entirely overcome the aftermath of this problematic period [14].

Since the early 90's a renewed interest in Surinamese gold exploitation has expanded gold mining activities, leading to an enormous migration into the various goldfields. Malaria became a serious problem for the people working and living in and around these fields. Until 2004, the situation within villages in the interior, which are mainly situated alongside the riverbanks, was a serious cause for concern as well.

### National malaria board

The call for an umbrella organization that could coordinate the fight against malaria in Suriname was made a long time ago. J.C. Panhuys wrote in 1943:

*"I wish for two of the most qualified health practitioners to be named extraordinary members of the council, and that a permanent Malaria-Board would be brought into life in which all medical staff would be seated" *[5].

Fifty-two years later his call was answered. The installation of the National Malaria Board, which was assembled in 1995, seems to be one of the factors responsible for the success of the current malaria control programme in Suriname. It consists of representatives from the Ministry of Health, Ministry of Regional Affairs, Ministry of Defense, the Bureau of Public Health, an infectiologist with specific expertise in malaria and the Medical Mission, a government-sponsored NGO responsible for primary health care in the whole interior of the country. All malaria cases in Suriname are currently registered by place of origin and entered in a weekly report that is reviewed by members of the National Malaria Board. The Board initiates prompt active case detection surveys when malaria re-emerges in a village that had been free of malaria or if the number of cases in a specific region increases abruptly. The cause of such an outbreak is then also investigated. The board is also initiator in obtaining adequate national and international funding for its projects. The "Malaria Champion of the Americas" award [15], received by the National Malaria Board of Suriname, was an initiative of the Pan American Health Organization (PAHO), Pan American Health and Education Foundation (PAHEF) and the George Washington University Center for Global Health (CGH) [16].

### MDG 2015 targets

Suriname has already exceeded its 2015 Millennium Development Goal (MDG) target for the reduction of malaria. It has halted and significantly reduced the incidence of malaria. Indicators of this MDG target are the prevalence and death rates associated with malaria and the proportion of the population in malaria-risk areas using effective malaria prevention and treatment measures. Malaria control is an important part of reaching the MDGs, especially in sub-Saharan Africa where the group most affected is children below five years of age - reducing malaria therefore also combats child mortality. In Suriname this strong correlation between child mortality and malaria can currently not be made any longer, since most malaria cases occur in the adult working population. The WHO goal of ending malaria deaths by 2015 appears to be realistic when it comes to Suriname, where in the last years virtually no malaria deaths seem to have occurred.

### Introduction of ACT

In Suriname, *Plasmodium falciparum *has been the most prevalent malaria species for a long time. This can partly be explained by the fact that most Maroon inhabitants of the inland regions, also called Bush Negroes - descendants of runaway slaves - lack the Duffy antigen, an erythrocyte surface receptor that *Plasmodium vivax *needs to invade red blood cells [17]. This Duffy antigen is also very uncommon in western and central Africa, which is the origin of most slaves that were brought to Suriname.

From 2001 increasing suspicion of resistance against quinine was raised in Suriname. At that time quinine was the first-line treatment for uncomplicated *P. falciparum *malaria in Suriname [18]. In response to this finding, five clinical efficacy trials were conducted between 2001-2006 in the capital city and the interior of Suriname, to evaluate six different treatment options that were available at that time, for their effectiveness and affordability [18]. Compliance with the five-day course of quinine had been poor and therefore constituted a major concern of Suriname's Ministry of Health. Unpleasant side effects were most likely responsible for the poor compliance. The low efficacy and poor tolerability of quinine that was found as a result of the trials led to a change of treatment policy. In January of 2004, the National Malaria Board advised the Ministry of Health to replace quinine with the combination therapy artemether-lumefantrine (AL) as first-line treatment of uncomplicated Plasmodium *falciparum *malaria in Suriname (Figure 2). The decision to make the switch to an ACT for the treatment of uncomplicated *P. falciparum *malaria was in accordance with WHO recommendations [19-21]. It proves to be highly effective at this stage, but the question is for how long this will be the case in view of first hints at emerging artemisinin resistance from South-East Asia [22].

### Drug resistance

Especially the Thai-Cambodian border has been a site of emerging anti-malarial drug resistance throughout history. In 2009, a clinical trial to evaluate treatment efficacy was conducted by Dondorp *et al. *in Pailin, Western Cambodia and Wang Pah, North-western Thailand. Results of this trial showed P. *falciparum *parasites with significantly reduced *in vivo *susceptibility to artesunate in Pailin as compared to the study group in Wang Pah [22]. The prolonged time to parasite clearance could not be explained by host factors or pharmacokinetic influences. Similar to the programme in Suriname, the Ministry of Health of Cambodia implemented an active malaria-control programme. The programme included the introduction of artemisinin-based combination therapies in 2001. Nonetheless, a recent survey, performed by Yeung *et al. *in 2008, showed that an excessively high number (78%) of artemisinin use in western Cambodia consisted of monotherapy instead of the advised combination therapy [23]. The private sector is the main provider of these monotherapies. This situation urgently calls for containment measures in order to limit the spread of resistant parasites. If artemisinin resistance would spread like previous anti-malarial-resistance did, this would be catastrophic for global malaria control [24].

Because of the low and even further declining incidence of malaria cases in Suriname, monitoring and evaluating current treatment efficacy is more difficult than it has been in the past. Limited numbers of patients are available to participate in clinical trials. During the writing of this paper, an AL single-arm efficacy trial was being carried out in Paramaribo (results unpublished to date).

### Global fund

In addition to the governmental financing, external funding is of great importance to the current success of the malaria control programme in Suriname. The institutions that are aiding the fight against malaria with international support are The Global Fund [25] and the Amazon Malaria Initiative (AMI) [26]. The Global Fund to fight Aids, Malaria and Tuberculosis has financed two major projects in Suriname.

### Local input, higher output

The first programme, initiated by the Medical Mission, started in 2006: *Decreasing the incidence of malaria in the populations of the interior of Suriname*. A network of clinics and community health workers was created to target people with little or no access to health care services. The main interventions of the programme were: Active case detection (ACD) campaigns, the distribution of nearly 70,000 long-lasting impregnated nets (LLINs), the protection of more than 5,000 individuals by additional indoor residual spraying (IRS) and information and communication meetings [27]. One of the interesting parts of the programme was the input of local communities with redesigning the insecticide-treated nets that were provided by the programme. Because the nets were especially designed to meet the needs of the users, the probability of local people using the nets increased.

### Specific target group

In recent years, since few malaria cases arise within villages in the interior of the country, mainly due to the project mentioned above, malaria transmission and major outbreaks of malaria in Suriname are almost exclusively related to transmission in the mobile population of people working in the gold mining industry. The second Global Fund programme, *'Looking for gold, finding malaria'*, started in 2008 and was implemented by the Bureau of Public Health. Creating a map of all the gold mining localities provided an important overview of the malaria problem areas. Because a significant proportion of the people working in the gold mines are illegal foreigners, the strategy of carrying out the programme without law enforcing institutions was essential for successful outreach. The health workers are trusted to only have interest in finding and treating malaria. The population itself is also actively involved in the treatment of malaria among its people. The use of Rapid Diagnostic Tests (RDTs) has proved to be essential, especially in remote areas where light-microscopy is not directly available. They reduce the time needed to make a definite diagnosis, which can be crucial in some situations. In addition these tests are easy to use, which created the possibility of assigning 'malaria service deliverers' (MDS's). In short, this means the training and equipping of lay individuals, for example cleaning ladies or shop owners, to provide prompt diagnosis and treatment of uncomplicated malaria. Smith et al. have described a similar approach [28]. They found that interventions aiming at informal providers showed great impact on behaviour, especially in the private sector. The training of shopkeepers and informal drug vendors, alongside with gaining their trust and cooperation is extremely important for the success of any programme trying to target the private sector of drug providers.

### Existing social structures

Reports dating back to the early seventies describe difficulties and successes in implementing preventive measures and treatment in Suriname [6]. Knowledge from previous research in the field of social science can prove to be very helpful in the management of programme implementation. Existing social structures must be capitalized on in order to increase the success of any health programme. Costs and rewards for the people participating need to be balanced. Therefore, cooperation with the group itself or native speakers can be essential for the programme to work. In this context, the fact that an important part of malaria infections originate across the border shows that cooperation with French Guyana and Brazil is extremely important - not only for patients but also for the Surinamese healthcare facilities who have to be familiar with working with the specific population of mainly Brazilian gold miners and accompanying professions. Because of this specific target group, the malaria diagnostic and treatment outpatient clinic located in the so-called Brazilian quarter of Suriname's capital city has proved to be very successful. With a native Brazilian working at the clinic, patients are able to communicate their thoughts and concerns with someone they can associate with. As one can imagine, the possibility to speak in one's native tongue is an important factor in any health care situation that must not be underestimated.

### Paradigm shift

A major difficulty exists in terms that this specific target population seems not overly concerned with malaria and appears to have accepted it as one of the risks associated with gold mining. The rapidly changing and migrating population needs to stay aware of the possible life threatening disease, and pass on their knowledge within the population. Nevertheless, even if the level of knowledge is sufficient, a change in knowledge does not automatically lead to a change in behaviour. To achieve this desired paradigm shift will be a huge challenge for the malaria control programme, MSD's and the target population in the following years.

### Asymptomatic malaria

Most malaria control programmes in the world are based on the examination and treatment of symptomatic cases, also known as passive case detection. Consequently, only people who suffer from symptoms and can take the time and effort to find health care will be diagnosed. Individuals who are not feeling ill may have persistent gametocytaemia and fuel on-going transmission. A different strategy is to actively search for malaria cases. This approach will result in discovering asymptomatic individuals as well. The Ministry of Health in Brazil has implemented the malaria control strategy of aggressive active case detection (AACD) since 1996 [29]. At the malaria diagnostic and treatment outpatient clinic in Paramaribo a certain part of asymptomatic cases are filtered out as well when people come to get tested out of precaution, to be certain of staying free of malaria when they return from the gold mining areas.

### Cooperation

Multiple cooperation bonds have been formed in the region of the Americas in order to combine forces in the fight against malaria. RAVReDA (Red Amazonica para la Vigilancia de la Resistencia a los Antimalaricos) is the Amazon Network for the Surveillance of Anti-malarial Drug Resistance. It is a network formed by seven South American countries: Bolivia, Brazil, Colombia, Ecuador, Guyana, Peru and Suriname. As part of the RAVReDA project that started in 2001, Suriname is monitoring drug resistance by periodically conducting clinical efficacy trials combined with molecular research. The malaria control programme in Suriname is currently challenged by the extensive use of informally provided, non-recommended anti-malarials and self-medication especially by the mining population [2]. RAVReDA's role in this issue is to monitor an eventual decline in treatment efficacy of the recommended anti-malarials and if so, investigate whether or not this could be due to emerging resistance to one or more of the treatment compounds. The strong cooperation with the malaria gene bank is an excellent example of molecular research supporting malaria policy of the Ministry of Health.

USAID's Latin America and Caribbean Bureau started the Amazon Malaria Initiative (AMI) in 2001. The AMI collaboration was made in order to address ineffective control and treatment of malaria. AMI's partner countries are the same as RAVReDA's. Currently all partner countries treat falciparum malaria with an ACT according to WHO recommendations. The third cooperation bond is formed between Suriname and French Guiana as part of the Transborder Health Project from France and Suriname. On the 21^st ^to the 23^rd ^of February 2011, Surinam hosted a Trans Border Malaria Meeting. Four countries, Brazil, France, Guyana and Suriname, participated in this meeting. During the meeting, each country presented current strategy and approaches for malaria control and elimination. Problem areas and challenges that national programmes encounter were discussed. In addition, workshops about various issues were organized in order to share visions, experiences and expertise. The participating countries decided to develop a malaria control cooperation that should involve the five countries of the Guyana Shield, thus Venezuela will also be invited to participate in this cooperation.

## Conclusions

Suriname has a long history of dealing with malaria. The efforts, struggle, setbacks and successes experienced throughout the years, have taught the health care facilities a great deal about the complex nature of controlling this disease. All the efforts made in the past have brought malaria in Suriname to the current stable situation of control and created the possibility of moving forward to elimination. These results must definitely be sustained. Therefore it is important to keep investing in the malaria control programme even if the situation seems under control. In 1943, J. C. Panhuys put this into words perfectly:

*"There seems to be only one solution: nonstop alertness of government and society. Even though, a lot will depend on the knowledge, precaution, insight and sacrifices of medical staff members" *[5].

This also refers to the fact that effective preventive measures and adequate therapeutic options are on their own not enough to control or eliminate malaria. Personal and social behaviour of people is crucial in making the efforts count. A main issue now is to keep people aware of malaria as well as the importance of keeping current treatment options available and effective. Enhancing knowledge of malaria in a mobile population residing in poorly accessible regions, let alone changing the behaviour of these people, is extremely challenging and, therefore, research about successfully implemented interventions and methods of measuring their effectiveness must be expanded. The following years will be crucial for Suriname and the Guyana Shield in showing whether or not the region can move forward to permanently eliminating malaria.

## Abbreviations

AACD: Aggressive active case detection; ACD: Active case detection; ACT: Artemisinin-based combination therapy; AL: Artemether-lumefantrine; AMI: Amazon Malaria Initiative; API: Annual parasite index; CGH: George Washington University Center for Global Health; DDT: Dichloro-diphenyl-trichloroethane; ENSO: El Niño Southern Oscillation; Global Fund: Global Fund to fight Aids: Malaria and Tuberculosis; IRS: Indoor residual spraying; ITNs: Insecticide-treated (bed)nets; KOTAB: K-O-Tab^® ^is a deltamethrin-based tablet for bed net impregnation; MDG: Millennium Development Goal; MEP: Malaria Eradication Programme; MSD: Malaria service deliverer; LLINs: Long-lasting insecticide-impregnated nets; PAHEF: Pan American Health and Education Foundation; PAHO: Pan American Health Organization; RAVReDA: Red Amazonica para la Vigilancia de la Resistencia a los Antimalaricos (Amazon Network for the Surveillance of Anti-malarial Drug Resistance); RDT: Rapid (malaria) diagnostic test; WHO: World Health Organization.

## Competing interests

The authors declare that they have no competing interests.

## Authors' contributions

FJVB did the literature search and wrote the first draft of the manuscript. SGSV supervised FJVB during her stay in Suriname and contributed to the writing of the final version of the manuscript. MPG conceived of the project, supervised FJVB in Amsterdam and contributed to the writing of the final manuscript. All authors approved of the final version of the manuscript.
